# A History of a Large Tumour, in the Region of the Abdomen, Containing Hair

**Published:** 1786

**Authors:** John Warren

**Affiliations:** Professor of Anatomy and Surgery in the University of Cambridge


X. *A History of a large Tumour, in the Region of the Abdomen, containing Hair. By* John Warren, *Esq. F. A. A. and M. S. and Professor of Anatomy and Surgery in the University of Cambridge.* — From the *Memoirs of the American Academy of Arts and Sciences,* Vol. I. 4to. Boston, 1785.

AN averfion to the making of large incifions into fuch tumours as have appeared to have been feated within the cavity of the abdomen, has perhaps often been the reafon why thofe of them which have happened to contain a fubftance lefs fluid than pus have either induced a hectic, from a copious abforption of the thinner and more acrid parts, or have fpeedily been followed by a fatal termination. The following hiftory may in fome meafure evince the fafety of fuch large and free openings in cafes of this kind; but the facts contained in it may alfo admit of an application to the purpofe of explaining certain phenomena in the animal œconomy.

The production of hair in the human body, though it has often been the fubject of accurate examination, and ingenious fpeculation, has perhaps never yet been fatisfactorily accounted for; or, to fay the leaft, the folution is ftill deftitute destitute of that support and conviction which, in most other physiological inquiries, have so happily been attained.

Admitting the position, that many, if not all the interior parts of the body are furnished with the necessary fluids for the growth of this substance, an accurate attention to the circumstances under which it is really produced, and to the nature of the parts in which it is most frequently found, must undoubtedly afford very considerable light on the subject; and from a large number of such facts, carefully and judiciously collected, it is not improbable that every doubt and difficulty attending the investigation may be entirely removed.

Z—— H——, a negro woman, about thirty-two years of age, early in the year 1783 applied for medical assistance in a case of a swelling in the abdomen, which had become extremely painful, and which began to be attended with very threatening symptoms. On examination, a very large tumour was found seated chiefly on the left side, occupying the whole space between the left os ilium and the left inferior ribs, and extending over to the right of the umbilical region, pointing a little to the left of the navel, considerably hard, and extremely fenfible. Upon inquiry into the origin of the tumour, it appeared, that the patient had firft complained of pain in the left groin, and a general enlargement of the abdomen, immediately after delivery of her third child: this gradually increafed after two fucceffive labours, and fince the birth of her laft child, now about twelve years of ago, had been almoft conftantly painful, though by no means in a very diftreffing degree, until about three weeks prior to her application for advice. At this period her complaints were greatly exafperated, in confequence of catching cold at the time of the catamenial evacuation, by which a total fuppreffion was induced. The common difcutient topical applications were immediately made ufe of; the ufual methods were employed to renew the difcharge; but all to no purpofe. The fwelling conftantly increafed for about three weeks, when an evident tendency to fuppuration being perceived, the method of cure was immediately altered from a difcutient to a fuppurative procefs. In about two weeks a fluctuation was perceptible, and at the end of two more an opening into the cavity of the tumour was determined upon.

An

An extenſive inciſion was accordingly made through the rectus muſcle, at a ſufficient diſtance from the uſual courſe of the epigaſtric artery to avoid all danger of wounding it, and about a pint of watery matter immediately iſſued through the orifice; after which about the ſame quantity of pure pus was diſcharged.

On introducing two or three fingers into the cavity, a quantity of ſoft ſubſtance was felt within it, much about the conſiſtency of ſoft ſope. I immediately made uſe of a table ſpoon as the moſt convenient inſtrument that could be readily procured for extracting it, and about a pound of it was at this time obtained; after which a degree of faintneſs began to enſue, the wound was dreſſed, and the patient placed in her bed in a proper ſituation for admitting of a free diſcharge of any fluid that might ſtill be retained.

At the three or four ſucceeding dreſſings a portion of the ſame ſubſtance was taken out, till the whole being extracted, it amounted to the quantity of about four pounds.

At each dreſſing the matter was particularly examined, and was found to contain a large quantity of ſhort hair or wool, about three quarters of an inch long, uniformly mixed with with it, as is feen in the fpecimen herewith prefented for the infpection of the Academy.

In each hair was difcoverable by the naked eye a bulbous root, and a pointed extremity, both perfectly fimilar to what is feen in an entire hair produced naturally in other parts of the body. After the removal of the whole fubftance, the hand was paffed into and round the cavity in fearch of bone, or any other foreign body which might be contained within it; but though fome of the gentlemen prefent, on fuppofition of an extra-uterine fœtus, expected to have found the former, yet nothing of either could be felt, though every part was fairly acceffible. From this examination it evidently appeared that the matter extracted had been contained in a fac which firmly adhered to the peritoneum, a circumftance, I believe, generally attendant on fuppurations in the vifcera of the abdomen, as the natural confequence of previous inflammation.

On being expofed to the heat of the fire in an open veffel, it emitted a ftrong urinous fmell, and was attended in other refpects with moft of the appearances ufually exhibited in the broiling of animal fubftances. The action of flame produced a fmart and continued decrepitation crepitation of the falt contained in it, until the whole was reduced to a fimple coal; but no figns of inflammability, or the prefence of any oily fubftance, were perceptible. When boiled, the water made ufe of in the procefs was very little changed as to its fenfible properties; but after ftanding fome time in a veffel to cool, it depofited a fediment which was fufpected to be an alkaline falt, and which accordingly readily fermented with the vitriolic acid.

The patient, from the ufe of the bark, fu-perficial dreffings, and a reftorative diet, was, in about three months, enabled to enter upon her ufual employment, which was bufinefs of the moft laborious kind. She had during her illnefs been much reduced, and for fome time continued in an emaciated ftate, yet fhe now enjoys perfect health, and has become mode-rately corpulent. The catamenial evacuation has been regularly performed; but no figns of pregnancy have ever appeared, though, before her ficknefs, fhe had borne children uncom-monly faft.

Many of the practitioners in the town of Bofton were called in to vifit the patient, and various were the conjectures upon the nature of the cafe. The abfence of all the ufual figns of of pregnancy; except at thofe periods which regularly preceded the refpective deliveries above mentioned, is a ftrong argument againft the hypothefis of an extra-uterine fœtus; and it fhould feem by no means admiffible that the bones fhould have been fo perfectly diffolved as to have formed with the mufcular, and other foft parts of a fœtus, one uniform and apparently homogeneous mafs of matter. Are we not authorized, from the general complexion of the cafe, particularly from the pain in the groin of the affected fide, to pronounce the ovarium to have been the fuffering part? The attachment of the ligamentum rotundum of the uterus to the adipofe fubftance in the groin, feems to point out, either the uterus, or fome other part connected with it, as the feat of the diforder.

A difeafed ovarium may eafily be conceived to acquire a fize too great to admit of its being contained in the pelvis, and from its elevation in the abdominal region, the uterus itfelf might alfo be raifed, and a diftention of the ligament thereby be produced. But it is farther probable, that from an immediate adhefion of the ovarium to the neighbouring part of the uterus, the inflammation with which thofe parts might be be affected would extend to the ligament itſelf; the former cauſe operating in conjunction with this, would ſufficiently account for the pain in the groin of the affected ſide; and the application of this reaſoning in the above inſtance, together with the other facts contained in the hiſtory, might enable us, without much difficulty, to form a pretty ſure diagnoſis of the diſeaſe.

